# Preparation and Efficacy Evaluation of Heat-Resistant Freeze-Dried Live-Attenuated Vaccine Formulation of *Micropterus salmoides* Rhabdovirus

**DOI:** 10.3390/vaccines14010106

**Published:** 2026-01-21

**Authors:** Hongru Liang, Guangwei Hu, Xia Luo, Qiang Lin, Xiaozhe Fu, Yinjie Niu, Baofu Ma, Wenwen Xiao, Zhengwei Cui, Ningqiu Li

**Affiliations:** 1 Key Laboratory of Fishery Drug Development, Ministry of Agriculture and Rural Affairs, Guangdong Province Key Laboratory of Aquatic Animal Immune and Sustainable Aquaculture, Pearl River Fisheries Research Institute, Chinese Academy of Fishery Sciences, Guangzhou 510380, China; 2 College of Oceanography, Fujian Agriculture and Forestry University, Fuzhou 350002, China

**Keywords:** *Micropterus salmoides* rhabdovirus, freeze-dried vaccine, thermostability, largemouth bass, immersion immunization

## Abstract

Background/Objectives: An attenuated strain of *Micropterus salmoides* rhabdovirus (MSRV) 0509 with good immunogenicity has been isolated, showing potential as a candidate for live vaccine development. Methods: To improve the shelf life of attenuated strain of MSRV0509, the virus was formulated using three distinct single-protectant formulations and twelve thermostable protective agent formulations (designated T1–T12). Following lyophilization, the thermostability of each formulation was evaluated. Results: Results indicated that formulations T1, T9, and T10 maintained stable viral titers after storage at 25 °C and 37 °C. Moreover, these formulations retained high viral viability after 12 months at 4 °C, with a titer reduction of less than 0.5 log_10_. Immunological analyses revealed that the freeze-dried MSRV vaccine elicited both humoral and immune factors responses in largemouth bass. Immersion immunization provided effective protection, yielding a survival rate exceeding 80%. Freeze-dried vaccines maintained their immunogenicity (i.e., the ability to induce antibodies) following 12 months of storage at 4 °C. Additionally, expression of IFN-γ and IL-12 was significantly upregulated in fish post-vaccination. Conclusions: In conclusion, the lyophilized MSRV vaccine developed in this study not only exhibits improved thermostability and extended shelf life, but also effectively preserves its immunogenic properties, supporting its potential for practical aquaculture applications.

## 1. Introduction

The largemouth bass (*Micropterus salmoides*) is a freshwater fish species originally native to the southeastern United States and later introduced to China [1], which has emerged as an economically significant species in Chinese aquaculture [2]. However, there are more and more diseases that pose significant challenges to largemouth bass, including *Micropterus salmoides* rhabdovirus (MSRV), largemouth bass virus (LMBV), and so on [3].

MSRV, a member of the family Rhabdoviridae, has caused great economic losses in China. The virus primarily affects juvenile fish, leading to clinical signs such as lethargy, abnormal swimming, and a bloated abdomen and distorted body shape, as well as induced necrotic ulceration and multi-organ hemorrhage [4], which could lead to a mortality rate of over 90%. Currently, there are no effective vaccines or antiviral therapies for MSRV infection [5].

In our previous study, an attenuated strain of MSRV0509 was isolated from largemouth bass and showed good immunogenicity, suggesting its potential as a live-attenuated vaccine candidate. MSRV is a single-stranded, negative-sense RNA virus enveloped by a lipid bilayer, which confers sensitivity to heat; the virus is rapidly inactivated at 56 °C. Like many biological products, vaccines are susceptible to degradation under suboptimal storage conditions, resulting in a limited shelf life, and gradually lose their potency [6]. Lyophilization is commonly employed to enhance the stability of live-attenuated vaccines [7].

The development of a stable freeze-dried formulation is essential for the practical application of live-attenuated vaccines in aquaculture. In this study, we aimed to (1) screen a range of protective agents for the freeze-drying of the MSRV0509 strain; (2) evaluate the long-term viability of the optimized formulation during storage at 4 °C; and (3) assess its tolerance to short-term temperature fluctuations. We successfully developed a freeze-dried vaccine that maintained high viability under long-term refrigerated storage and exhibited robustness against transient temperature changes. These findings provide critical data for establishing the expiration date and recommended storage conditions of the MSRV-attenuated freeze-dried vaccine, thereby supporting its future commercial use in aquaculture disease prevention.

## 2. Materials and Methods

### 2.1. Viruses and Reagents

The Chinese perch brain cell line (CPB) was used for MSRV0509 propagation [8] and was grown in Leibovitz’s L-15 medium (L-15; Gibco, New York, NY, USA) supplemented with 8% fetal calf serum (FBS; HyClone, Logan, UT, USA) at 28 °C. The MSRV0509 was obtained from the Pearl River Fishery Research Institute (Guangzhou, China). Largemouth bass, with a body length of 2.5 ± 0.5 cm and an average weight of 0.5 ± 0.02 g, were obtained from an MSRV-free zone in FOSHAN (Foshan, China). *Polyethylene glycol* (PEG) 4000, N-Z-amine, sucrose, and gelatin were purchased from Med Chem Express (Shanghai, China).

### 2.2. MSRV0509 Propagation

Monolayer cultures of CPB cells were infected with MSRV0509 at a multiplicity of infection (MOI) of 0.0001. After 1 h of adsorption, the cells were washed with PBS to remove unabsorbed virus, and L15 culture medium was added to a final concentration of 2% fetal bovine serum, with the temperature shifted to 28 °C. When more than 90% of the cells show cytopathic effects, the virus was harvested and 50% tissue culture infective doses (TCID_50_ titers) were determined using the Reed–Muench method [9].

### 2.3. Preparation of Single-Protective Agent Formulations

To assess the efficacy of traditional single-protective agent formulations, MSRV0509 vaccines containing an individual protective agent were prepared. Specifically, solutions of 1% skim milk powder, 2% gelatin, and 8% sucrose were prepared as single protective agents. Each agent was sterilized under high pressure and subsequently mixed uniformly with MSRV0509 at a 1:1 ratio. The freeze-drying procedure consisted of three stages: 2 h at −50 °C, 24 h at −40 °C, and 20 h at −25 °C; the formulations were lyophilized at a rate of 0.5 °C/min and the vacuum pressure was maintained at <10 Pa. The vaccine bulk was filled into 10 mL type glass vials at a volume of 1.0 mL per vial.

### 2.4. Preparation of Heat-Resistant Protective Agent Formulations

To evaluate the efficacy of heat-resistant protective agent formulations, MSRV0509 vaccines containing various heat-resistant protective agents were prepared. Twelve distinct heat-resistant protective agent formulations (TT-12) were prepared as shown in Table 1, which were composed of 10~20% sucrose, 5~15% N-Z-amine, and 1~3% PEG 4000, among other components. Agent A, which contained sucrose and gelatin, underwent high-pressure sterilization, while Agent B, containing N-Z-amine and PEG, was filter-sterilized. Agents A and B were mixed with MSRV-0509 in a 1:1:1 ratio. The freeze-drying procedure consisted of three stages: 2 h at −50 °C, 24 h at −40 °C, and 20 h at −25 °C. The final formulations were aliquoted into penicillin bottles for freeze-drying.

### 2.5. Evaluation of Key Properties of Freeze-Dried Vaccines

#### 2.5.1. Scanning Electron Microscopy (SEM) Observation of the Freeze-Dried Vaccine

To examine the microscopic morphology of the freeze-dried vaccine, samples from different formulations were observed using a scanning electron microscope (SEM) (ZEISS, Oberkochen, Germany). The analysis aimed to characterize and compare the structural features of the lyophilized products.

#### 2.5.2. Moisture Residue Analysis (RM)

To determine the moisture residue content in various formulations of freeze-dried vaccines, samples of each formulation were transferred into pre-weighed weighing bottles, and their initial mass was recorded. The bottles were then placed in a vacuum-drying oven and dried for 3 h at 65 °C. After drying, the bottles were reweighed to assess the mass loss.

Remaining moisture = (weight before sample drying − weight after sample drying)/weight before sample drying × 100%.

#### 2.5.3. Thermal Stability of Different Freeze-Dried Vaccines

To assess the thermal stability of the different freeze-dried vaccine formulations, sealed vials of each formulation were stored in temperature-controlled incubators at two distinct temperatures: 25 °C and 37 °C. Throughout the stability study, samples (*n* = 3 per formulation per time point) were aseptically withdrawn from each incubator every day. Immediately after collection, these samples were rapidly transferred and stored at −80 °C. Titers of all samples were determined using the Reed–Muench method (TCID_50_).

#### 2.5.4. Shelf Life Testing of Freeze-Dried Vaccines

To determine the shelf life of different freeze-dried vaccine formulations, sealed vials of each formulation were stored in temperature-controlled incubators at two distinct temperatures: −20 °C and 4 °C. and samples (*n* = 3 per formulation per time point) were aseptically withdrawn from each incubator every month. Immediately after collection, these samples were rapidly transferred and stored at −80 °C. Titers of all samples were determined using the Reed–Muench method (TCID_50_) [9].

### 2.6. Freeze-Dried Vaccine Immunization and Clinical Observation

To evaluate the safety experiment of freeze-dried vaccines, largemouth bass underwent immersion immunization with different concentrations of different freeze-dried vaccines, as follows: (1) The freeze-dried vaccine was reconstituted in PBS (phosphate-buffered saline) to a concentration of 10^4^ TCID_50_/mL. (2) The freeze-dried vaccine was reconstituted in PBS to a concentration of 10^5^ TCID_50_/mL. (3) The freeze-dried vaccine was reconstituted in PBS to a concentration of 10^6^ TCID_50_/mL. Fish were immunized by immersion in different concentrations solution for one hour and subsequently transferred to a tank for feeding. (4) PBS was used as a negative control, and fish were immersed in PBS for one hour under identical immunization conditions before being placed in a tank for feeding. Clinical signs of each group (*n* = 35) were monitored daily for 14 days.

### 2.7. Fish Immune Protection Experiment

To evaluate the immune protection conferred by freeze-dried vaccines in largemouth bass, the following experiments were conducted after 12 months of storage at 4 °C: (1) The freeze-dried vaccine was reconstituted in PBS to a concentration of 10^5^ TCID_50_/mL. Fish were immunized by immersion in this solution for one hour and subsequently transferred to the tank for feeding. (2) A non-lyophilized liquid formulation of MSRV0509, adjusted to 10^5^ TCID_50_, served as the positive control. Fish were immunized by one-hour immersion and then moved to the tank for feeding. (3) PBS was used as a negative control, and fish were immersed in PBS for one hour under identical immunization conditions before being placed in the tank for feeding. Twenty-one days post-immunization, each group of largemouth bass (*n* = 60) was challenged intraperitoneally with MSRV-QY at a dose of 50 LD_50_. Clinical signs were monitored daily for 14 days following the challenge.

### 2.8. Detection of Antibodies by Viral Neutralization Assay

To detect specific antibodies in largemouth bass following immersion immunization, blood samples were collected from three fish per group. The experiment was repeated three times under the same conditions. Serum was separated, and neutralization titers were determined using a viral neutralization assay. The procedure was performed as follows: (1) We diluted the serum in a 2-fold series. (2) A volume of 50 μL of each diluted serum was mixed with 50 μL of MSRV0509 virus containing 10 TCID_50_, and the mixture was incubated at room temperature for 1 h. (3) The serum–virus mixture was added to a monolayer of CPB cells and incubated at room temperature for 1 h. (4) After adding 100 μL of L15 medium, the cells were maintained at 28 °C, with all conditions tested in triplicate. The cytopathic effect (CPE) was evaluated for cells observed under a microscope after 10 days.

### 2.9. Detection of Immune Factors by Quantitative Reverse Transcription Polymerase Chain Reaction (RT-qPCR)

To detect the expression of immune factors in largemouth bass after immersion immunization, the spleen and head kidney were sampled at various time points. Six samples were taken from each group at each time point, and the experiment was repeated three times under the same conditions. Total RNA was extracted using the Viral RNA Extraction Kit (TRANS, Beijing, China), and complementary DNA (cDNA) was synthesized by reverse transcription with TranScript cDNA Synthesis SuperMix (TRANS, China), in accordance with the manufacturer’s instructions. RT-qPCR was subsequently performed using PerfectStar Green qPCR SuperMix (TRANS, China) on an Applied Biosystems 7500 instrument. The amplification protocol consisted of initial denaturation at 94 °C for 30 s, followed by 40 cycles of denaturation at 94 °C for 5 s and annealing/extension at 60 °C for 15 s. Expression levels of IFNγ (NCBI: XM_038709291.1) and IL-12 (NCBI: XM_038708060.1) were quantified using the following gene-specific primers: IFNγ: forward, 5′-GGAGTTGCTTTGGCGTTTG-3′; reverse, 5′-GTCGTGCTCATTGTGGCTGT-3′; IL-12: forward, 5′-TCTTCCATCCTTGTGGTCTTCC-3′; reverse, 5′-CAGTTCCAGGTCAAAGTG GTC-3′. The 18S rRNA gene was used as an internal reference based on its common application in studies of teleost immune gene expression [10]. The 18S rRNA gene was used as an internal reference. The calculated efficiencies for all genes (IFNγ, IL-12, and 18S rRNA) were between 90% and 110%, and the difference in efficiency between target and reference genes was less than 5%, meeting the prerequisite for valid use of the 2^−ΔΔCt^ method [11] and thus validating its use for relative quantification. Relative gene expression was calculated using the 2^−ΔΔCt^ method.

### 2.10. Statistical Analysis

The survival outcomes were analyzed using Kaplan–Meier survival analysis. The relative expression levels of immune-related genes across vaccinated groups were analyzed using one-way ANOVA (GraphPad Prism 8.0). Other results are reported as mean ± standard error (SE). Data met the assumptions for parametric testing (normality and homogeneity of variance). Statistical comparisons were performed with two-tailed Student’s *t*-tests, and significance was evaluated against thresholds of *p* < 0.05 (*) and *p* < 0.01 (**).

## 3. Results

### 3.1. Characterization of Freeze-Dried Vaccines

#### 3.1.1. Physical Characterization of Freeze-Dried Vaccine Formulation

**Figure 1:**
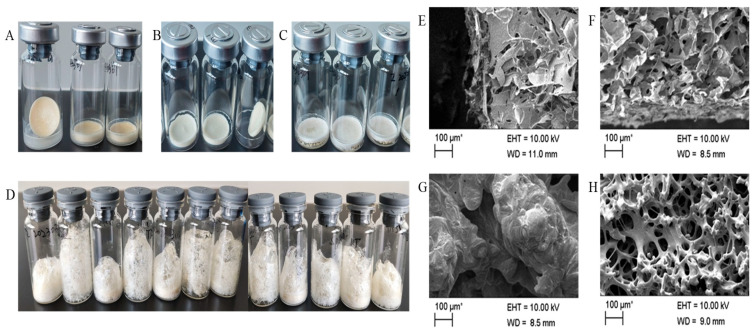
Physical characterization of freeze-dried vaccine formulation. Formulations containing a single protective agent: skim milk (**A**), gelatin (**B**), and sucrose (**C**); (**D**) formulations with thermostable protective agents: T1~T12; (**E**) SEM of formulations containing a single protective agent: skim milk; (**F**) SEM of formulations containing a single protective agent: gelatin; (**G**) SEM of formulations containing a single protective agent: sucrose; (**H**) SEM of formulations with thermostable protective agents.

Significant structural differences were observed in various formulations of freeze-dried vaccines. Formulations containing a single protective agent (e.g., sucrose, gelatin, or milk) exhibited a relatively compact and dense morphology (Figure 1A–C). In contrast, formulations prepared with thermostable protective agents (T1–T12) displayed a highly porous, foamy structure characterized by numerous bubbles, resulting in a stable dry foam matrix after lyophilization (Figure 1D).

The microscopic morphology of the freeze-dried vaccine formulations was examined using scanning electron microscopy (SEM). At higher magnification, skim milk- and gelatin-based formulations showed flake-like structures organized in a regular lamellar pattern, accompanied by numerous small, dense aggregates (Figure 1E,F). Sucrose-containing samples, however, revealed an irregular sponge-like structure (Figure 1G). Formulations incorporating heat-resistant stabilizers consistently formed a porous network with regular reproducible structural features (Figure 1H). All samples demonstrated clearly defined physical characteristics and exhibited consistent reconstitution behavior in aqueous solution.

#### 3.1.2. Moisture Residue Analysis (RM)

**Figure 2:**
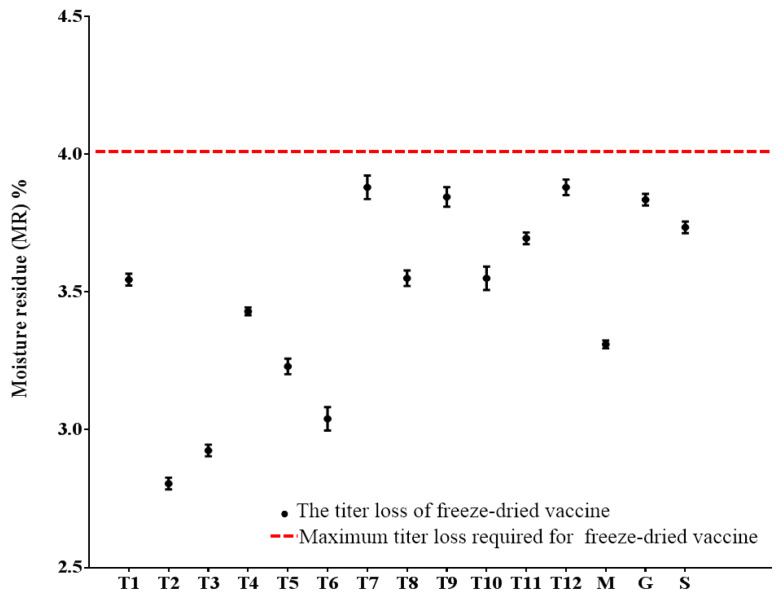
Moisture residue analysis (RM). (T1–T12): Formulations with thermostable protective agents; (M): formulations containing a single protective agent: skim milk; (G): formulations containing a single protective agent: gelatin; (S): formulations containing a single protective agent: sucrose.

The moisture residue content of the freeze-dried vaccine formulations was determined (Figure 2), all formulations exhibited moisture residue levels below 4%, which was within the acceptable limit prescribed for biological products. These results confirm that the lyophilization process was effective in achieving low moisture content across all tested formulations, thereby meeting the critical quality attribute for storage stability.

#### 3.1.3. Screening of Freeze-Dried Formula for MSRV Live-Attenuated Vaccine

**Figure 3:**
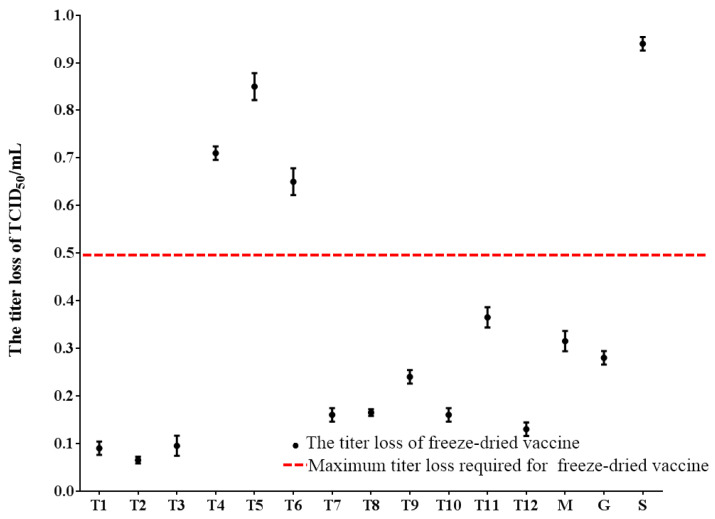
The virus titer loss of freeze-dried formula for MSRV live-attenuated vaccine. (T1–T12): Formulations with thermostable protective agents; (M): formulations containing a single protective agent: skim milk; (G): formulations containing a single protective agent: gelatin; (S): formulations containing a single protective agent: sucrose. The protective efficacy of the lyophilized formulations was evaluated by comparing the viral titers before and after freeze-drying. Formulations T4, T5, T6, and S were excluded from subsequent analyses due to a post-lyophilization titer loss exceeding the threshold of 0.5 Log_10_.

The protective efficacy of the lyophilized formulations was evaluated by comparing the viral titers before and after freeze-drying. As illustrated in Figure 3, the freeze-drying process induced varying degrees of viral titer reduction across the formulations, indicative of variable structural and functional damage to the viral components. Formulations T4, T5, T6, and S were excluded from subsequent analyses due to a post-lyophilization titer loss exceeding the threshold of 0.5 Log_10_, a predefined criterion for acceptable viability loss.

#### 3.1.4. Thermal Stability of Different Freeze-Dried Formulas for MSRV Live-Attenuated Vaccine

**Figure 4:**
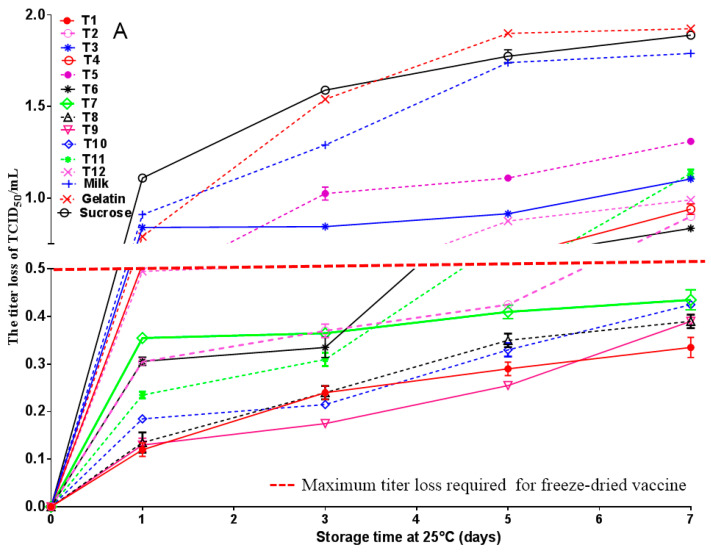

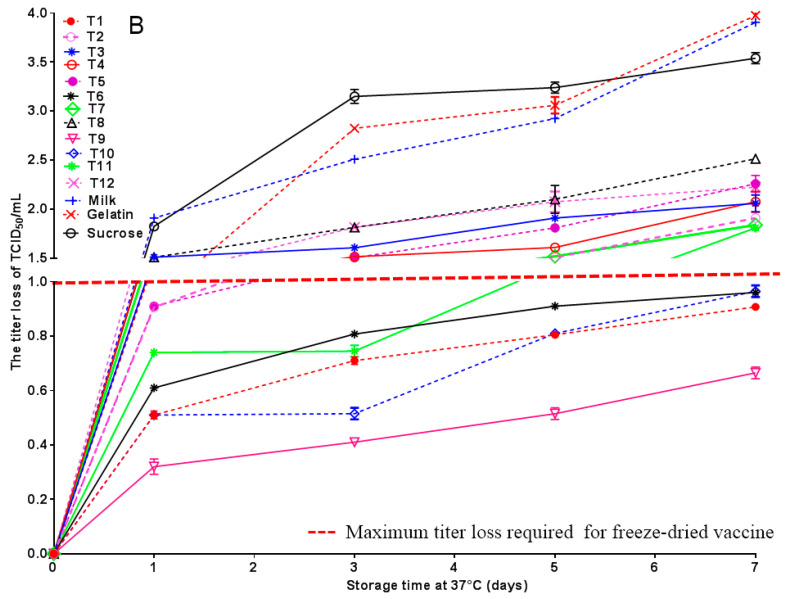
Thermal stability of different freeze-dried formulas for MSRV live-attenuated vaccine. The thermal stability of the freeze-dried vaccine formulations was evaluated by incubating samples at 25 °C (**A**) and 37 °C (**B**). Formulations T1, T9, and T10 consistently maintained acceptable viral viability under both temperature conditions and were identified as the most stable configurations.

The thermal stability of the freeze-dried vaccine formulations was evaluated by incubating samples at 25 °C and 37 °C, followed by measurement of viral titers. After storage at 25 °C for 7 days, formulations T1, T7, T8, T9, and T10 demonstrated superior stability, with a titer loss not exceeding 0.5 Log_10_ (Figure 4A). Similarly, upon incubation at 37 °C for 7 days, formulations T1, T6, T9, and T10 exhibited improved thermal resistance, showing titer reductions within 1.0 Log_10_ (Figure 4B). Based on these criteria, formulations T1, T9, and T10 consistently maintained acceptable viral viability under both temperature conditions and were identified as the most stable configurations. These results suggest that the excipient composition of these formulations contributes significantly to enhancing the thermostability of the lyophilized vaccine.

#### 3.1.5. Shelf Life Stability of Freeze-Dried Vaccines

To assess the shelf life stability of the freeze-dried vaccine formulations, samples were stored under two conditions: −20 °C and 4 °C. Viral titers were periodically monitored over a 12-month period. Formulations containing a single protective agent exhibited a rapid decline in viral titer during storage at both −20 °C and 4 °C. A significant reduction in potency was observed as early as 6 months, indicating their unsuitability for long-term vaccine preservation.

**Figure 5:**
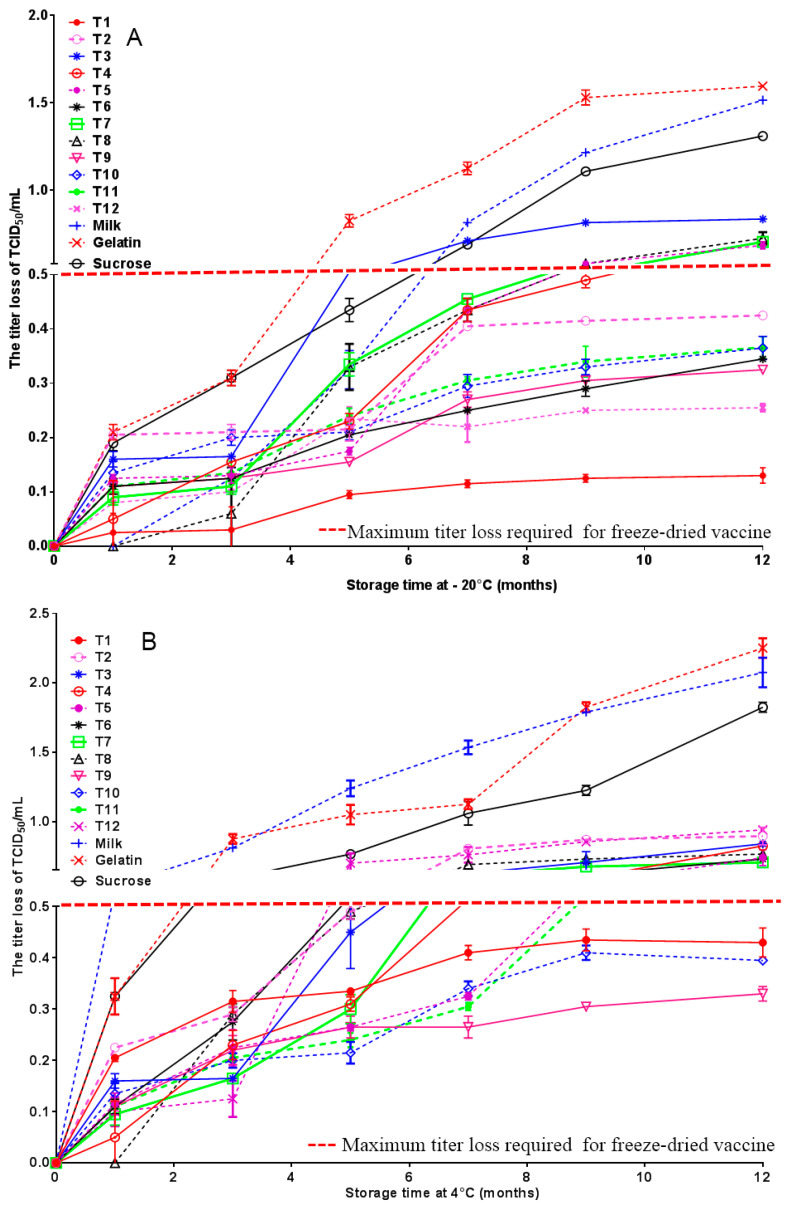
Shelf life stability of freeze-dried vaccines. The shelf life stability of the freeze-dried vaccine formulations was evaluated by detected viral titers for 12 months at −20 °C (**A**) and 4 °C (**B**) T1, T9 and T10 provide enhanced long-term thermostability, making them promising candidates for the development of commercially viable vaccines with reduced cold-chain dependency.

In contrast, formulations comprising heat-resistant protective agents (T1, T2, T6, T9, T10, T11, T12) demonstrated markedly improved stability. After 12 months of storage at −20 °C (Figure 5A), these formulations maintained viral titers with a loss not exceeding 0.5 Log_10_. Furthermore, a subset of these formulations (T1, T9, T10) also exhibited excellent stability at 4 °C, with titer losses similarly remaining below 0.5 Log_10_ after 12 months (Figure 5B). These results indicate that the optimized formulations, particularly T1, T9, and T10, provide enhanced long-term thermostability, making them promising candidates for the development of commercially viable vaccines with reduced cold-chain dependency.

### 3.2. Safety Experiment of Freeze-Dried Vaccines

All groups of fish were observed for 14 days, and the survival rate of all the groups was 100%.

### 3.3. Fish Immune Protection Experiment

**Figure 6:**
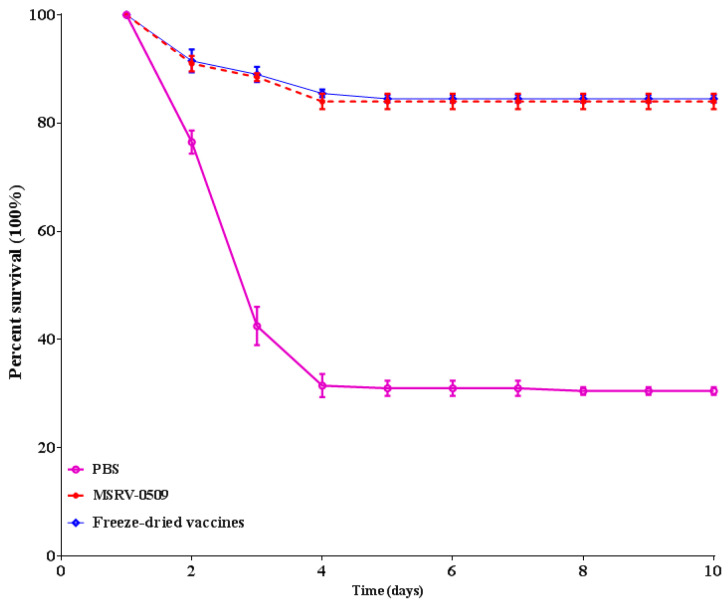
Fish immune protection experiment. Largemouth bass were immunized via immersion with either the liquid MSRV0509 vaccine or the freeze-dried formulation of T9 to assess the immune-protective efficacy of the freeze-dried vaccine. The results were analyzed using Kaplan–Meier survival analysis. The survival rates in vaccinated groups exceeded 85%, whereas the survival rate in the negative control group was only 30%. The results indicate that the liquid MSRV0509 vaccine and freeze-dried protective agents have protective effects on vaccines.

Largemouth bass were immunized via immersion with either the liquid MSRV0509 vaccine or the freeze-dried formulation of T9. The results demonstrated that both the liquid and freeze-dried vaccines conferred significant protection against subsequent challenge. The survival rate with liquid vaccines was 85% and that with freeze-dried vaccines was 86%, whereas the survival rate in the negative control group was only 30% (Figure 6). These findings indicate that the freeze-drying process did not compromise the immunogenic properties of the vaccine and that the lyophilized formulation provides protective immunity comparable to that of its liquid counterpart.

### 3.4. Detection of Neutralizing Antibodies by Viral Neutralization Assay

To evaluate the humoral immune response induced by vaccination, serum samples were collected from largemouth bass following immersion immunization with either the liquid MSRV0509 vaccine or the freeze-dried formulation. Virus-neutralizing antibody titers in the serum were quantified using a viral neutralization assay conducted on CPB cells.

**Figure 7:**
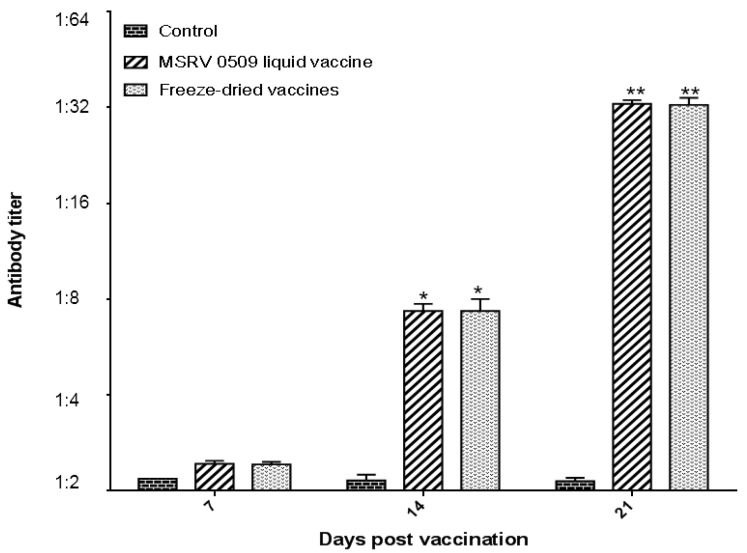
Detection of neutralizing antibodies by viral neutralization assay. Control: fish were immersed in PBS; MSRV0509 liquid vaccine: fish were immersed in a non-lyophilized liquid formulation; freeze-dried vaccines: fish were immersed in T9 freeze-dried vaccines. Serum samples were collected from largemouth bass following immersion immunization with either the liquid MSRV0509 vaccine or the freeze-dried formulation. The results indicated that serum from both the liquid and freeze-dried vaccine groups effectively neutralized the virus, as evidenced by the absence of cytopathic effect (CPE) at serum dilutions ranging from 1:2 to 1:32. Statistical significance is indicated as follows: * *p* < 0.05, ** *p* < 0.01. Data are presented as means ± SE (*n* = 3).

The results indicated that serum from both the liquid and freeze-dried vaccine groups effectively neutralized the virus, as evidenced by the absence of cytopathic effect (CPE) at serum dilutions ranging from 1:2 to 1:32 (Figure 7). Notably, the freeze-dried vaccine stored at 4 °C for 12 months elicited neutralizing antibody titers comparable to those induced by the liquid vaccine. These findings demonstrate that the freeze-dried vaccine retains its immunogenicity and ability to provoke a potent virus-neutralizing antibody response in fish following long-term storage.

### 3.5. Detection of Immune Factors by RT-qPCR

**Figure 8:**
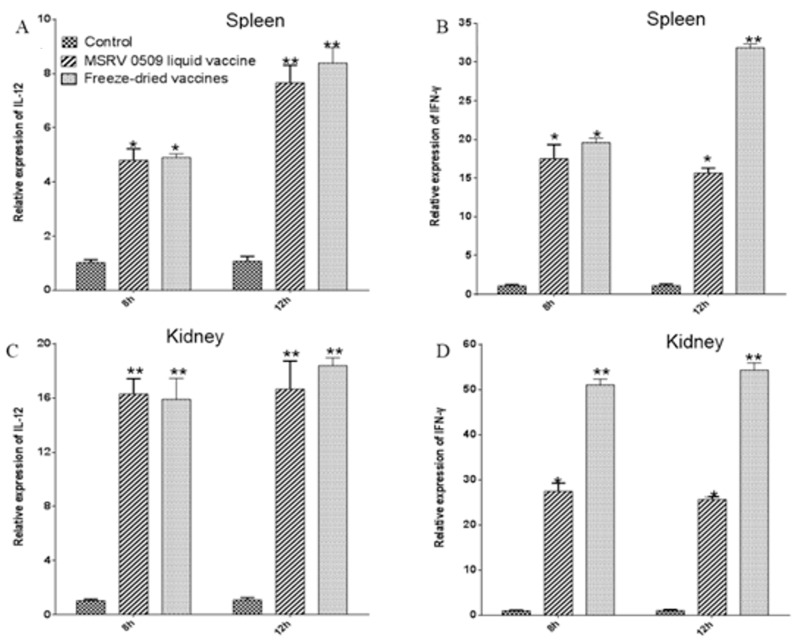
Detection of immune factors by qRT-PCR. Relatvie expression of IL-12 in spleen (**A**) and kidney (**C**); Relatvie expression of IFN-γ in spleen (**B**) and kidney (**D**). Spleen and head kidney tissues were collected from largemouth bass at various time points post-vaccination to investigate the innate immune response induced by immersion immunization. There was a rapid upregulation of both IFN-γ and IL-12 transcripts as early as 8 h post-immunization in groups administered either the liquid MSRV0509 vaccine or the freeze-dried formulation, compared to pre-immune controls. Statistical significance is indicated as follows: * *p* < 0.05, ** *p* < 0.01. Data are presented as means ± SE (*n* = 3).

To investigate the innate immune response induced by immersion immunization, each group of nine spleen and head kidney tissues was collected from largemouth bass at various time points post-vaccination. The results revealed a rapid upregulation of both IFN-γ and IL-12 transcripts as early as 8 h post-immunization in groups administered with either the liquid MSRV0509 vaccine or the freeze-dried formulation, compared to pre-immune controls (Figure 8). This early induction suggests activation of pro-inflammatory and Th1-type immune pathways following vaccination. Notably, the freeze-dried vaccine elicited a gene expression profile comparable to that of the liquid vaccine, indicating that the lyophilization process preserved the immunostimulatory properties of the formulation.

## 4. Discussion

The largemouth bass *(Micropterus salmoides)*, introduced to China in 1983, has become an economically significant aquaculture species [2]. However, the intensive cultivation of this species has led to frequent outbreaks of infectious diseases caused by bacteria and viruses, posing serious threats to industry sustainability [12]. *Micropterus salmoides* rhabdovirus (MSRV) is particularly detrimental to juvenile fish, with a reported mortality rate of 90% [13]. Although compounds such as baicalein and dihydroartemisinin have shown some protective effects in challenged fish [14,15], vaccination remains the most effective strategy for controlling viral diseases in aquaculture [16,17].

Freeze-drying (lyophilization) is a widely used technique for improving the stability of biological products by removing water through sublimation under low-temperature and vacuum conditions [18]. It is commonly employed in the production of commercial live vaccines [19], which significantly extends shelf life by converting the product into a solid form with minimal moisture residue [20]. However, the process can induce protein denaturation and aggregation, primarily due to the removal of structural water during primary and secondary drying, leading to a loss of biological activity upon reconstitution [21].

The formulation of lyophilized products strongly depends on the selection of appropriate excipients to enhance stability and cost-effectiveness [22]. Stabilizers such as sugars, surfactants, and salts are often incorporated to protect protein integrity during freeze-drying [23]. Sugars can act as cryoprotectants by forming hydrogen bonds with proteins, effectively replacing water molecules and maintaining structural stability. Additionally, certain excipients like polyethylene glycol (PEG) exhibit surface-active properties that help suppress protein aggregation in solid formulations. Similarly, components such as N-Z-amine have been found to reduce aggregate formation at higher concentrations.

In this study, we formulated live-attenuated MSRV vaccines using PEG 4000, Gelatin, sucrose, and NZA as key stabilizers. N-Z-Amine is an enzymatic digest of casein, which helps to buffer against the dramatic increases in solute concentration and pH shifts that can damage the virus during freezing. Gelatin is a natural polymer derived from the partial hydrolysis of collagen, used as a stabilizer in biologics and vaccines. Three single-protectant and twelve thermostable formulations were prepared and lyophilized. All freeze-dried vaccines demonstrated rapid reconstitution properties and moisture residue content below 4%, consistent with pharmacopeial standards for biological products.

One of the methods used in potency evaluation of live vaccines is determining the titer of the vaccine [6]. Based on the results of appearance, virus titer, and thermal stability tests after freeze-drying, the most suitable formula (T1, T9, and T10) is selected for freeze-dried protectants. The titer of single-protective formulations decreases rapidly in the thermal stability test and shelf life test. Formulations (T1, T9, and T10) showed good heat resistance, which inhibited the infectivity loss within 0.5 log_10_ and indicated that they had a better stabilized than others. Hence, the vaccine formulation in T1, T9, and T10 improved the stability and long-term storage of the live-attenuated MSRV vaccine.

Juvenile largemouth bass measuring approximately 3~5 cm in length are particularly susceptible to MSRV infection. Fish fry in the seedling stage are too small for injection immunization; therefore, soaking immunization is a preferred immune pathway. Immersion vaccination represents a practical approach for large-scale immunization in aquaculture, particularly for small fish, as it mimics natural pathogen entry and can induce mucosal immunity. Immersion vaccination is a particularly cost effective method of administration in very small fish [24]. In the challenge, experiments demonstrated that juvenile largemouth bass vaccinated via immersion with the T9 lyophilized vaccine (stored for 12 months at 4 °C) achieved a survival rate exceeding 80%, significantly higher than the 30% observed in the control group. Moreover, serum antibody levels in fish immunized with the lyophilized vaccine were comparable to those induced by the liquid formulation, confirming the retention of immunogenic potency after long-term storage.

Notably, elevated expression of key cytokines such as IFN-γ and IL-12 post-vaccination suggests robust activation of both innate and cellular immune pathways. This is particular importance in early antiviral defense, given the delayed adaptive immune response in fish [25]. Cytokines such as interleukin-1 (IL-1) and interleukin-6 (IL-6) trigger the expression of a suite of serum proteins that orchestrate a protective host response, aimed at minimizing self-injury and directly neutralizing pathogens [8]. In this study, the results showed that IFN-γ and IL-12 were significantly upregulated after immersion infection by MSRV0509 liquid vaccines and freeze-dried vaccines. Therefore, the freeze-dried MSRV0509 vaccine induced a response involving both humoral and immune factors in largemouth bass. As the findings are currently limited to the mRNA level, future work should be directed toward validating these gene expression changes at the protein level—for example, through proteomics or cellular assays—to confirm their functional significance.

## 5. Conclusions

In conclusion, we have developed a lyophilized MSRV vaccine using optimized stabilizer formulations that significantly enhance thermostability and shelf life without compromising immunogenicity. The vaccine elicits strong immune responses and provides effective protection against MSRV challenge. These results support the potential of this freeze-dried product as a viable candidate for large-scale vaccination in sustainable largemouth bass aquaculture.

## Figures and Tables

**Table 1:** Composition of the various formulations used for the MSRV live-attenuated vaccine.

Formulation Code	Composition			
	NZA (% *w*/*v*)	Sucrose (% *w*/*v*)	PEG (% *w*/*v*)	Gelatin (% *w*/*v*)
T1	5%	10%	1.5%	1.5%
T2	5%	15%	1.0%	1.5%
T3	5%	10%	1.0%	1.5%
T4	5%	15%	1.5%	1%
T5	5%	10%	1.5%	1%
T6	5%	15%	1.0%	1%
T7	10%	10%	1.5%	1.5%
T8	10%	15%	1.5%	1.5%
T9	10%	10%	1.5%	1%
T10	10%	15%	2.0%	1%
T11	10%	10%	2.0%	1%
T12	10%	20%	1.5%	1%

PEG indicates polyethylene glycol; NZA, N-Z-amine.

## Data Availability

The data that support the findings of this study are available from the corresponding author upon reasonable request.
